# Serologic Prevalence of Amoeba-Associated Microorganisms in Intensive Care Unit Pneumonia Patients

**DOI:** 10.1371/journal.pone.0058111

**Published:** 2013-03-01

**Authors:** Sabri Bousbia, Laurent Papazian, Pierre Saux, Jean-Marie Forel, Jean-Pierre Auffray, Claude Martin, Didier Raoult, Bernard La Scola

**Affiliations:** 1 Aix Marseille Université, URMITE, UM63, CNRS 7278, IRD 198, Inserm 1095, Marseille, France; 2 Service de Réanimation Médicale, Hôpital Nord, Marseille, France; 3 Département d’Anesthésie-Réanimation, Hôpital de la Timone, Marseille, France; 4 Département d’Anesthésie-Réanimation, Hôpital Nord, Marseille, France; Institut de Pharmacologie et de Biologie Structurale, France

## Abstract

**Background:**

Patients admitted to intensive care units are frequently exposed to pathogenic microorganisms present in their environment. Exposure to these microbes may lead to the development of hospital-acquired infections that complicate the illness and may be fatal. Amoeba-associated microorganisms (AAMs) are frequently isolated from hospital water networks and are reported to be associated to cases of community and hospital-acquired pneumonia.

**Methodology/Principal Findings:**

We used a multiplexed immunofluorescence assay to test for the presence of antibodies against AAMs in sera of intensive care unit (ICU) pneumonia patients and compared to patients at the admission to the ICU (controls). Our results show that some AAMs may be more frequently detected in patients who had hospital-acquired pneumonia than in controls, whereas other AAMs are ubiquitously detected. However, ICU patients seem to exhibit increasing immune response to AAMs when the ICU stay is prolonged. Moreover, concomitant antibodies responses against seven different microorganisms (5 *Rhizobiales*, *Balneatrix alpica*, and Mimivirus) were observed in the serum of patients that had a prolonged ICU stay.

**Conclusions/Significance:**

Our work partially confirms the results of previous studies, which show that ICU patients would be exposed to water amoeba-associated microorganisms, and provides information about the magnitude of AAM infection in ICU patients, especially patients that have a prolonged ICU stay. However, the incidence of this exposure on the development of pneumonia remains to assess.

## Introduction

Hospital-acquired infections are systematically reported as one of the most important causes of mortality in hospitalized patients [1]–[4]. Hospital-acquired pneumonia (HAP) is a leading infection in intensive care units (ICUs) and it is frequently associated with high rates of mortality and morbidity [4]. It is a serious risk complicating the illness, especially for patients undergoing mechanical ventilation for which it is estimated that approximately 7% to 70% develop ventilator-associated pneumonia.

Amoeba-associated microorganisms (AAMs) are microorganisms that may multiply and resist to destruction by free-living amoebae. Thus amoebae are considered as potential reservoir of these microorganisms. Waterborne pathogens that are associated with free-living amoebae such as *Parachlamydia* spp., *Chlamydia* spp., *Afipia* spp., *Bosea* spp. and *Acanthamoeba polyphaga* mimivirus (Mimivirus) are potential pathogens that can infect patients admitted to ICUs and may be the etiology of ICU-acquired pneumonia [5]–[10]. Previous studies have showed that these amoeba-resistant microorganisms can be isolated from hospital water sources and environmental water [9], [11]–[14].

The diagnostic tools that are usually employed to isolate the etiologic pathogen of pneumonia include standard cultures of respiratory samples and blood cultures. However, these diagnostic tools cannot identify most of fastidious microorganisms such as some amoeba-associated microorganisms (AAMs). Amoeba-associated co-culture is an alternative pathway to identify these microorganisms. Such a powerful diagnostic approach is very time consuming for samples that are furthermore frequently contaminated with oro-pharyngeal flora. Serological tests, like the immunofluorescence assay (IFA), represent an attractive alternative that can be used to analyze samples rapidly for epidemiologic studies. However, it is technologically challenging to simultaneously analyze samples that have a complex mixture of antigens. The multiplexed serologic assay (i.e., microarray serology) has recently been shown to be an efficient serologic diagnostic tool that can simultaneously analyze a variety of microorganisms in a single experiment. This technique can be used to study a complex mixture of several pathogens in one disease such as hospital-acquired pneumonia (HAP) [15]–[17].

In this study, we assessed the prevalence of amoeba-associated microorganisms in sera from ICU patients and more in particular in sera from pneumonia patients. The majority of the examined patients were undergoing mechanical ventilation and many of them developed one or more episodes of pneumonia during their stay in the ICU.

## Results

### Prevalence of Antibodies to Microorganisms

In order to study the association of AAMs with pneumonia, we tested the frequency of these AAMs in a control cohort (admission sera) and compared it to their frequency in an ICU-pneumonia cohort. In total, we collected 173 serum samples from 97 patients: 29 admission serum samples, 88 acute phase pneumonia serum samples (55 ventilator-associated pneumonia sera, 17 community-acquired pneumonia sera, 8 aspiration pneumonia sera and 8 non-ventilator ICU pneumonia sera) and 56 weekly serum samples. In pneumonia cohort, acute respiratory distress syndrome (ARDS) was diagnosed in 36 patients (41%) (6 patients with community-acquired pneumonia (CAP); 25 patients with ventilator-associated pneumonia (VAP); 2 patients with aspiration pneumonia (AP) and 3 patients with non-ventilator ICU pneumonia (NV-ICU-P)). In this cohort, 24 (27%) were immunocompromised (6 patients with CAP, 17 patients with VAP and 1 patient with AP).

In both controls and pneumonia cohorts, the prevalence of IgM antibody response to AAMs was higher than the IgG antibody response. The frequencies of AAMs in controls and acute phase of pneumonia are listed in Table 1. In controls, for AAMs, IgM antibody response was most frequently detected against *B. massiliensis* (9 sera, 31%), whereas an IgG antibody response was most frequently detected against *B. liaoningense* (8 sera, 28%). No antibodies to *B. eneae* and Rasbo bacterium were detected. For non-AAMs, IgM antibody response was most frequently detected against *B. alpica* (5 sera, 17%), whereas no seroreactivity to *C. pneumoniae*, *C. psittaci* and *M. pneumoniae* was present (Table 1). Controls may exhibit IgM antibody response against up to 9 microorganisms in a single serum (mean ± SD, 1.68±2.49), while IgG antibody response were detected against up to 8 microorganisms in a single serum (mean ± SD, 0.62±0.82).

**Table 1:** Prevalence of antibodies to amoeba-associated microorganisms in pneumonia and in control (admission) sera.

Antigen	Admission sera (n = 29)		Pneumonia sera (n = 88)	
	IgG	IgM	IgG	IgM
AAMs				
Water Alpha-Proteobacteria :				
*Afipia birgiae*	0	1 (3%)	0	2 (2%)
*Afipia broomeae*	0	1 (3%)	0	1 (1%)
*Afipia felis*	1 (3%)	1 (3%)	4 (5%)	6 (7%)
*Afipia felis* genospecies A	0	2 (6%)	5 (6%)	13 (14%)
*Afipia* genospecies 1	0	2 (6%)	0	7 (8%)
*Afipia* genospecies 2	0	3 (10%)	0	8 (9%)
*Afipia* genospecies 3	0	4 (14%)	7 (8%)	18 (20%)
*Afipia massiliae*	0	2 (6%)	0	6 (7%)
*Afipia saintantoinensis*	0	2 (6%)	0	5 (6%)
*Azorhizobium caulinodans*	0	3 (10%)	1 (1%)	11 (13%)
*Bosea eneae*	0	0	3 (3%)	9 (10%)
*Bosea massiliensis*	1 (3%)	9 (31%)	1 (1%)	37 (42%)
*Afipia quartiernordensis*	1 (3%)	0	0	2 (2%)
*Bosea vestrisii*	0	1 (3%)	2 (2%)	8 (9%)
*Bosea thiooxidans*	0	1 (3%)	0	6 (7%)
*Bradyrhizobium japonicum*	2 (7%)	0	8 (9%)	2 (2%)
*Bradyrhizobium liaoningense*	8 (28%)	1 (3%)	25 (28%)	2 (2%)
Mesorhizobium amorphae*	1 (3%)	2 (6%)*	1 (1%)	20 (23%)*
*Nordella oligomobilis*	1 (3%)	4 (14%)	1 (1%)	26 (30%)
Rasbo bacterium	0	0	0	6 (7%)
Chlamydiae:				
*Parachlamydia acanthamoeba* BN9	0	1 (3%)	7 (8%)	10 (11%)
Water viruses:				
Mimivirus	1 (3%)	3 (10%)	9 (10%)	9 (10%)
Non-AAMs				
*Afipia clevelandensis*	0	1 (3%)	3 (3%)	0
*Balneatrix alpica*	2 (7%)	5 (17%)	9 (10%)	29 (33%)
*Chlamydia pneumoniae*	0	ND	8 (9%)	ND
*Chlamydia psittaci*	0	ND	0	ND
*Mycoplasma pneumoniae*	ND	0	ND	1 (1%)

ND; not determined. The asterisk (*) indicates a statistically significant frequency.

In acute phase pneumonia samples, for AAMs, the most frequent antibody response was against water alpha-Proteobacteria (Table 1). An IgM antibody response was frequently observed against *M. amorphae* (20 sera, 23%) *Afipia* genospecies 3 (18 sera, 20%), *B. massiliensis* (37 sera, 42%) and *N. oligomobilis* (26 sera, 30%). IgG antibody response was most frequently detected against *B. liaoningense* (25 sera, 28%). For non-AAMs, IgM antibody response was most frequently detected against *B. alpica* (29 sera, 33%), whereas no immune response to *C. psittaci* was found. In this cohort, IgM antibody response against up to 12 microorganisms were detected in a single patient serum (mean ± SD, 2.77±3.33), while IgG antibody response were detected for up to 5 microorganisms in a single serum (mean ± SD, 1.06±1.24). There was an IgM antibody response for at least one microorganism in 32 sera (36%), whereas an IgG antibody response was negative in 40 sera (45%). The anti-AAM antibody frequencies in patients with each type of acute phase pneumonia are shown in Table 2.

**Table 2:** Prevalence of antibodies to amoeba-associated microorganisms in acute phase sera from patients with various types of pneumonia.

Antigen	Pneumonia sera (acute phase; n = 88)							
	VAP sera (n = 55)		CAP sera (n = 17)		AP sera (n = 8)		NV-ICU-P sera (n = 8)	
	IgG	IgM	IgG	IgM	IgG	IgM	IgG	IgM
AAMs								
Water Alpha-Proteobacteria :								
*Afipia birgiae*	0	0	0	1 (6%)	0	1 (12%)	0	0
*Afipia broomeae*	0	0	0	0	0	1 (12%)	0	0
*Afipia* genospecies 1	0	3 (5%)	0	2 (12%)	0	1 (12%)	0	1 (12%)
*Afipia* genospecies 2	0	4 (7%)	0	2 (12%)	0	2 (25%)	0	0
*Afipia* genospecies 3	6 (10%)	11 (20%)	0	5 (29%)	0	2 (25%)	1 (12%)	0
*Afipia felis*	3 (5%)	4 (7%)	0	1 (6%)	0	1 (12%)	1 (12%)	0
*Afipia felis* genospecies A	5 (10%)	9 (16%)	0	2 (12%)	0	1 (12%)	0	1 (12%)
*Afipia massiliae*	0	2 (4%)	0	2 (12%)	0	2 (25%)	0	0
*Afipia quartiernordensis*	0	0	0	1 (6%)	0	1 (12%)	0	0
*Afipia saintantoinensis*	0	1 (2%)	0	3 (17%)	0	1 (12%)	0	0
*Azorhizobium caulinodans*	1 (2%)	7 (12%)	0	2 (12%)	0	1 (12%)	0	1 (12%)
*Bosea eneae*	2 (4%)	7 (12%)	1 (0%)	2 (12%)	0	0	0	0
*Bosea massiliensis*	0	26 (47%)	0	6 (35%)	0	3 (37%)	1 (12%)	2 (25%)
*Bosea thioxydans*	0	3 (5%)	0	0	0	2 (25%)	0	1 (12%)
*Bosea vestrisii*	1 (2%)	2 (4%)	1 (6%)	3 (17%)	0	1 (12%)	0	2 (25%)
*Bradyrhizobium japonicum*	6 (10%)	1 (2%)	1 (6%)	0	0	0	1 (12%)	1 (12%)
*Bradyrhizobium liaoningense*	14 (25%)	1 (2%)	5 (29%)	0	2 (25%)	0	4 (50%)	1 (12%)
*Mesorhizobium amorphae*	1 (2%)	15 (27%)	0	3 (17%)	0	1 (12%)	0	1 (12%)
*Nordella oligomobilis*	1 (2%)	16 (29%)	0	5 (29%)	0	2 (25%)	0	3 (37%)
*Rasbo bacterium*	0	3 (5%)	0	0	0	2 (25%)	0	1 (12%)
Chlamydiae:								
*Parachlamydia acanthamoeba* BN9	6 (10%)	5 (10%)	1 (6%)	4 (23%)	0	1 (12%)	0	0
Water viruses:								
Mimivirus	5 (10%)	7 (12%)	2 (12%)	2 (12%)	0	0	2 (25%)	0
Non-AAMs								
*Afipia clevelandensis*	2 (4%)	0	1 (6%)	0	0	0	0	0
*Balneatrix alpica*	7 (12%)	21 (38%)	0	4 (23%)	0	2 (25%)	2 (25%)	2 (25%)
*Chlamydia pneumoniae*	5 (10%)	ND	3 (17%)	ND	0	ND	0	ND
*Chlamydia psittaci*	0	ND	0	ND	0	ND	0	ND
*Mycoplasma pneumoniae*	ND	0	ND	0	ND	0	ND	1 (12%)

VAP, ventilator-associated pneumonia; CAP, community-acquired pneumonia; AP, Aspiration pneumonia and NV-ICU-P, Non ventilator ICU-acquired pneumonia, ND; not determined.

By comparing the frequency of anti-AAM antibodies in acute phase pneumonia sera and control sera, our results revealed that antibodies (IgG or IgM, or both) to *A. felis*, *A. felis* genospecies A, *Afipia* genospecies 1, *Afipia* genospecies 3, *B. eneae*, *B. massiliensis*, *B. vestrisii*, *B. thiooxidans*, *M. amorphae*, *N. oligomobilis*, Rasbo bacterium, *C. pneumoniae*, *P. acanthamoeba* BN9 were found more frequent in sera from the acute-phase of pneumonia than in control sera (Table 1). However, statistical analysis showed that only the frequency of anti- *M. amorphae* IgM antibodies was statistically significant (p = 0.05). Anti-microbial antibodies to *A. birgiae*, *A. broomeae*, *Afipia* genospecies 2, *A. massiliae*, *A. saintantoinensis*, *A. caulinodans*, *A. quartiernordensis*, *B. japonicum* and *B. liaoningense* were commonly detected at nearly the same prevalence in controls as well as in pneumonia sera (Table 1).

### Prevalence of Anti-AAMs Antibodies in Control Sera Versus the Convalescent Phase Sera

Immune reactivity may be latent and antibodies to AAMs may not be detected in the acute phase of pneumonia. Thus, in order to study a probable association between anti-AAM antibodies and HAP, we compared the anti-AAM antibody frequency of the admission sera (controls) to that of sera from convalescent patients after an episode of HAP. Our results show that antibodies to five AAMs and to one non-amoeba water bacterium were significantly more frequently found found in the convalescent-phase sera after HAP episode compared to the admission sera (Table 3). An IgG antibody response was more frequently detected against *A. felis* genospecies A, *Afipia* genospecies 3 and *P. acanthamoebae* BN9 in the convalescent phase sera compared to the admission sera (p = 0.05, p = 0.03 and p = 0.03, respectively).Similarly, serum from the convalescent phase were significantly more frequently exhibited an IgM response against *B. eneae* and *M. amorphae* compared to the admission sera (p = 0.01 and p = 0.02, respectively) (Table 3).

**Table 3:** Prevalence of antibodies to amoeba-associated microorganisms in convalescent-phase sera after hospital-acquired pneumonia and in control (admission) sera.

Antigen	Admission sera (n = 29)		Convalescent phase (n = 41)		P-value	
	IgG	IgM	IgG	IgM	IgG	IgM
AAMs						
Water Alpha-Proteobacteria :						
*Afipia birgiae*	0	1 (3%)	0	0		
*Afipia broomeae*	0	1 (3%)	0	0		
*Afipia* genospecies 1	0	2 (6%)	0	1 (2%)		
*Afipia* genospecies 2	0	3 (10%)	1 (2%)	3 (7%)		
*Afipia* genospecies 3	0	4 (14%)	6 (15%)	11 (27%)	**0.03**	
*Afipia felis*	1 (3%)	1 (3%)	7 (17%)	4 (10%)		
*Afipia felis* genospecies A	0	2 (6%)	5 (12%)	8 (20%)	**0.05**	
*Afipia massiliae*	0	2 (6%)	0	1 (2%)		
*Afipia quartiernordensis*	1 (3%)	0	0	0		
*Afipia saintantoinensis*	0	2 (6%)	0	2 (5%)		
*Azorhizobium caulinodans*	0	3 (10%)	0	4 (10%)		
*Bosea eneae*	0	0	3 (7%)	7 (17%)		**0.01**
*Bosea massiliensis*	1 (3%)	9 (31%)	1 (2%)	16 (39%)		
*Bosea thiooxidans*	0	1 (3%)	0	4 (10%)		
*Bosea vestrisii*	0	1 (3%)	0	3 (7%)		
*Bradyrhizobium liaoningense*	8 (28%)	1 (3%)	14 (34%)	2 (5%)		
*Bradyrhizobium japonicum*	2 (7%)	0	5 (12%)	0		
*Mesorhizobium amorphae*	1 (3%)	2 (6%)	0	12 (29%)		**0.02**
*Nordella oligomobilis*	1 (3%)	4 (14%)	0	12 (29%)		
*Rasbo bacterium*	0	0	0	3 (7%)		
Chlamydiae:						
*Parachlamydia acanthamoeba* BN9	0	1 (3%)	6 (15%)	6 (15%)	**0.03**	
Water viruses:						
Mimivirus	1 (3%)	3 (10%)	7 (17%)	9 (22%)		
Non-AAMs						
*Afipia clevelandensis*	0	1 (3%)	1 (2%)	0		
*Balneatrix alpica*	2 (7%)	5 (17%)	6 (15%)	18 (44%)		**0.01**
*Chlamydia pneumoniae*	0	ND	4 (10%)	ND		
*Chlamydia psittaci*	0	ND	0	ND		
*Mycoplasma pneumoniae*	ND	0	ND	1 (2%)		

ND; not determined.

### Seroconversion Rates to AAMs

Overall, high rates of seroconversion were observed in sera recovered from 7 days to greater than 3 months after admission. Anti-AAM IgM antibody seroconversion was more frequently observed than IgG seroconversion. Seroconversion was more frequent for the water Proteobacteria and Mimivirus (Table 4). IgM seroconversion to at least one microorganism was observed in 38 (53%) patients, while IgG seroconversion was observed in 34 (47%) patients. IgM seroconversion for up to 14 microorganisms may be detected in a single serum (mean ± SD, 3.39±2.95), whereas IgG seroconversion for up to 4 microorganisms was detected in a single serum (mean ± SD, 1.73±0.99). However, except for *Mycoplasma pneumoniae*, for which seroconversion was shown only after pneumonia episodes (after one CAP episode and one HAP episode), seroconversion to AAMs was found in patients who exhibited pneumonia as well as in patients who did not exhibit pneumonia but had an ICU stay longer than 7 days. We next analyzed the serology data for evidence of a class switch from IgM to IgG. Class switch represents a maturation of the immune response and thus provides evidence for active immune recognition of the target. Our findings showed that IgM to IgG class switching was frequent against *A. felis*, *A. felis* genospecies A, *B. liaoningense* and Mimivirus (Table S1). However, when we compared IgM to IgG class switching in pneumonia serum samples to these from patients without pneumonia, no microorganism showed a statistically significant difference and thus did not provide evidence for an association with pneumonia.

**Table 4:** Seroconversion rates to amoeba-associated microorganisms.

Antigen	Seroconversion rates							
	After CAP (n = 9)		After HAP (n = 48)		Without pneumonia (n = 14)		Total (n = 71)	
	IgG	IgM	IgG	IgM	IgG	IgM	IgG	IgM
AAMs								
Water Alpha-Proteobacteria :								
*Afipia birgiae*	0	0	0	0	0	1(7%)	0	1(1%)
*Afipia broomeae*	0	0	0	0	0	1(7%)	0	1(1%)
*Afipia felis*	0	0	6(13%)	4(8%)	1(7%)	2(14%)	7(10%)	6(8%)
*Afipia felis* genospecies A	2(22%)	1(11%)	3(6%)	4(8%)	0	1(7%)	5(7%)	6(8%)
*Afipia* genospecies 1	0	0	0	3(6%)	0	1(7%)	0	4(6%)
*Afipia* genospecies 2	0	0	1(2%)	2(4%)	0	1(7%)	1(1%)	3(4%)
*Afipia* genospecies 3	1(11%)	1(11%)	2(4%)	6(13%)	1(7%)	4(29%)	4(6%)	11(15%)
*Afipia massiliae*	0	0	0	2(4%)	0	1(7%)	0	3(4%)
*Afipia quartiernordensis*	0	0	0	0	1(7%)	0	1(1%)	0
*Afipia saintantoinensis*	0	0	0	2(4%)	0	1(7%)	0	3(4%)
*Azorhizobium caulinodans*	0	0	0	3(6%)	0	3(21%)	0	6(8%)
*Bosea eneae*	0	1(11%)	1(2%)	2(4%)	0	2(14%)	1(1%)	5(7%)
*Bosea massiliensis*	0	0	2(4%)	6(13%)	0	2(14%)	2(3%)	8(11%)
*Bosea thiooxidans*	0	0	0	3(6%)	1(7%)	3(21%)	1(1%)	6(8%)
*Bosea vestrisii*	0	0	0	2(4%)	0	1(7%)	0	3(4%)
*Bradyrhizobium japonicum*	2(22%)	0	1(2%)	0	1(7%)	0	4(6%)	0
*Bradyrhizobium liaoningense*	0	0	8(17%)	3(6%)	2(14%)	3(21%)	10(14%)	6(8%)
*Mesorhizobium amorphae*	0	1(11%)	0	5(10%)	0	5(36%)	0	11(15%)
*Nordella oligomobilis*	2(22%)	0	0	6(13%)	0	2(14%)	3(4%)	8(11%)
Rasbo bacterium	0	0	0	4(8%)	0	4(29%)	0	8(11%)
Chlamydiae:								
*Parachlamydia acanthamoeba* BN9	0	0	4(8%)	4(8%)	2(14%)	0	6(8%)	4(6%)
Water viruses:								
Mimivirus	2(22%)	1(11%)	4(8%)	5(10%)	0	2(14%)	6(8%)	8(11%)
Non-AAMs								
*Afipia clevelandensis*	0	0	0	0	0	0	0	0
*Balneatrix alpica*	2(22%)	1(11%)	3(6%)	11(23%)	0	5(36%)	5(7%)	17(24%)
*Chlamydia pneumoniae*	0	ND	3(6%)	ND	1(7%)	ND	4(6%)	ND
*Chlamydia psittaci*	0	ND	0	ND	0	ND	0	ND
*Mycoplasma pneumoniae*	ND	1(11%)	ND	1(2%)	ND	0	ND	2(3%)

CAP, community-acquired pneumonia; HAP, Hospital-acquired pneumonia; ND; not determined.

### Impact of ICU Stay on AAM Infection

**Figure 1:**
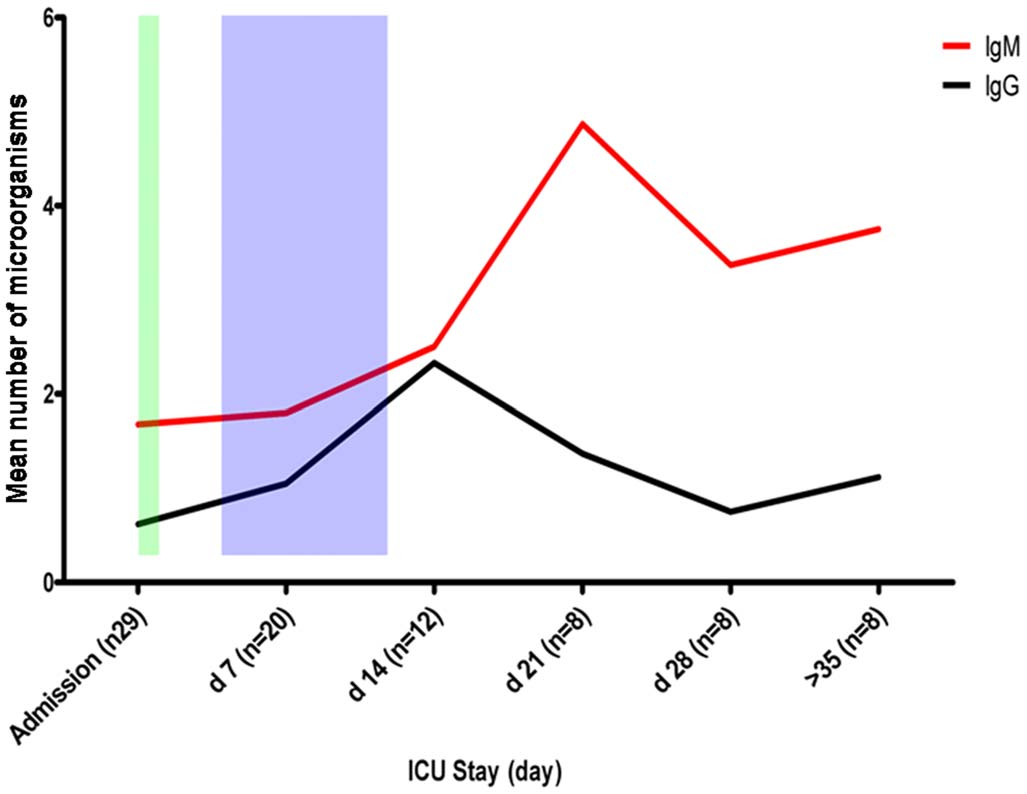
The association between a prolonged ICU stay and exposure to amoeba-associated microorganisms. The blue period corresponds to the period where a majority of the hospital-acquired pneumonia episodes (>95%) occurred. The CAP episodes mainly occurred from 12 to 24 hours after admission (green period). d; day.

To study the effects of a prolonged ICU stay on exposure to AAMs, we separately computed mean number of microorganisms against which the patients exhibited an antibody response for admission serum samples (n = 29) and for samples collected every 7 days thereafter (weekly serum samples; n = 56). Data indicate that the number of organisms against which patients (with or without pneumonia) exhibit an immune response (essentially with IgM antibodies) is positively correlated with the length of the patient ICU stay (Fig. 1). Overall, our patients developed an antibody response to an increasing number of AAMs over time, especially from days 7 to 21 following their admission (Fig. 1). These findings highlight the high seroconversion rates exhibited by ICU patients during their stay (Table 4) and confirm that immune response to AAMs increases when the ICU stay is prolonged.

### Hierarchical Cluster Analysis of Serologic Data

**Figure 2:**
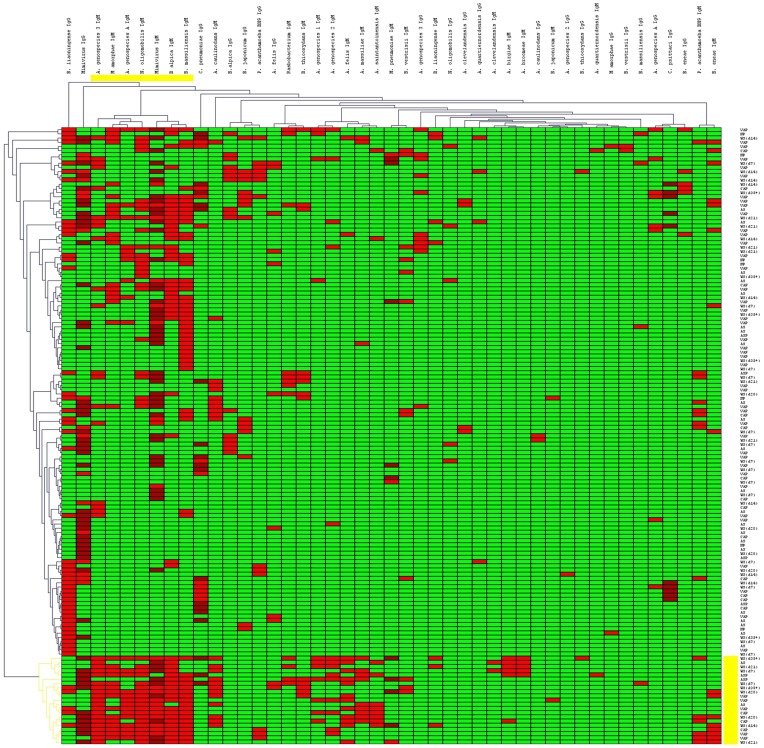
Hierarchical cluster analysis of patient antibody response to amoeba-associated microorganisms. This evaluation helped us to identify clusters of microorganisms that are specific to each patient group. To perform this analysis, T-Mev software was used. In the software, number “3” was attributed to positive sera and represented by red color. Number “−3” was attributed to negative sera and represented by green color. Number “2” was attributed to the low-positive sera and represented by dark red sera color. The main cluster detected is shown in yellow. It includes *Afipia* genospecies 3, *M. amorphae, A. felis* genospecies A, *N. oligomobilis,* Mimivirus, *B. alpica* and *B. massiliensis*. (AS, admission sera; WS, weekly sera; VAP, ventilator-associated pneumonia sera; CAP, community-acquired pneumonia sera; ASP, Aspiration pneumonia sera; NP, Non ventilator ICU pneumonia sera).

Hierarchical cluster analysis was used to group sera exhibiting similar antibody responses. This evaluation helped us to identify clusters of microorganisms specific to each patient group. To perform this analysis, T-Mev software was used. In the software, number “3” was attributed to positive sera and represented by red color. Number “−3” was attributed to negative sera and represented by green color. Number “2” was attributed to the low-positive sera and represented by dark red sera color. Results show the presence of a cluster associated to IgM antibody response to seven microorganisms (*Afipia* genospecies 3, *M. amorphae, A. felis* genospecies A, *N. oligomobilis,* Mimivirus, *B. alpica* and *B. massiliensis*) (yellow cluster in Fig. 2). These microorganisms were mainly found in patient sera collected from the acute phase of VAP and CAP, as well as in these from convalescent patients who had a prolonged ICU stay (Fig. 2).

## Discussion

Amoeba-associated microorganisms (AAMs) are fastidious organisms that grow optimally at 30 to 35°C. These microorganisms are extremely difficult to isolate with conventional culture media. They are frequently isolated from hospital water sources, and previous studies have shown that they are associated with community- and hospital-acquired pneumonia [9]. However, there are very few cohort-comparative studies about the association between AAMs and pneumonia in hospitalized patients. Recently, application of multiplexed array technology to human serum has allowed researchers to analyze large numbers of microorganisms in a high-throughput manner. This method is extremely reliable, rapid and reproducible [17]–[22]. We used microarray serology to analyze a panel of AAMs in ICU patients who are exposed to potentially contaminated hospital water sources. Our findings show that some AAMs may be more frequently detected after pneumonia episodes than in controls, whereas others are ubiquitous. The results of the comparison of AAMs frequency in convalescence phase sera and in control sera are partially concordant to previous studies and show that an association between AAMs and HAP may exist. In a previous study reported by La Scola et al., numerous alpha-Proteobacteria, especially *Afipia*, *Bosea*, *Bradyrhizobium* and *Mesorhizobium* species, were isolated from water samples collected from ICUs. Samples were collected with an amoebal co-culture procedure and frequency of antibodies against *Afipia* genospecies 2, *B. massiliensis* and *M. amorphae* was found to be significantly higher in patients with pneumonia compared to controls [9], [13]. In particular, *B. massiliensis* was significantly associated with a high seroconversion rate after VAP episodes. Furthermore, its DNA was detected in the broncho-alveolar lavage of a patient with pneumonia who later seroconverted [9]. An antibody response against this bacterium was again observed in another patient with pneumonia [7]. In our patients, *M. amorphae* was more frequently found in sera from patients with pneumonia, and from convalescent patients than in controls (p = 0.05 and p = 0.02 for IgM antibodies, respectively), which indicates that exposure to this bacterium is associated with pneumonia. Similarly, *A.* genospecies 3, *A. felis* genospecies A and *B. eneae* were frequently found in sera of patients during a convalescent phase after pneumonia (p = 0.03 and p = 0.05 for IgG antibodies to *A.* genospecies 3 and *A. felis* genospecies A, respectively, and p = 0.01 for IgM antibodies to *B. eneae*). However, our pneumonia patients exhibited significant antibody titers against *A.* genospecies 3, *A. felis* genospecies A and *B. eneae* but not against *A.* genospecies 2 and *B. massiliensis,* as already reported in previous studies by La Scola et al. (Table 3). This discrepancy may be due to higher serological cross-reactivity within the same bacterial species, which has been previously reported [23], or to the different geographical location of the ICUs or to seasonal differences in the sampling period. Our results show that some *Bosea* and *Afipia* species may be associated with HAP. However, such association remains to be confirmed by further future studies.

Other serological studies have reported high antibody titers against *P. acanthamoebae* in patients with CAP, VAP or aspiration pneumonia. Thus, the bacterium was thought to cause pneumonia [5], [24]–[27]. *P. acanthamoebae* is a rare Chlamydia-like bacterium which has been isolated by amoeba co-culture from water networks. In our patients, a seropositive reaction to the *P. acanthamoebae* BN9 antigen was significantly higher in the convalescent-phase sera compared to the admission sera (p = 0.03) (Table 3). Similarly, *C. pneumoniae* was serodiagnosed in 12 acute phase sera (3 sera from CAP and 5 sera from VAP) and 4 convalescent phase sera, but not in admission sera. These findings suggest that these two bacteria can be the etiological agent.

Among amoeba-associated viruses [28], [29], Mimivirus is the only one that has been associated with pneumonia [7], [8]. In a recent study by Vincent et al., a positive antibody response against Mimivirus was associated with a longer ICU stay and mechanical ventilation [29], [30]. Similarly, our study shows that seroconversion to Mimivirus was observed in 14 (20%) out of 71 patients with paired serum samples. Of these patients, seroconversion was observed in 79% of patients 14 days after admission. Additionally, an antibody response (both IgG and IgM) to Mimivirus antigens was increased in the convalescent-phase sera compared to the admission sera (4 out of 29 admission sera were positive for Mimivirus versus 16 out of 41 convalescent phase sera, p = 0.02). Our results suggest that this virus may play a role in HAP pathogenesis. This hypothesis is supported by previous studies showing that this virus is able to experimentally induce episodes of acute pneumonia [29], [31].

In addition to the amoeba-associated microorganisms, five non amoeba-associated bacteria (*A. clevelandensis, B. alpica*, *Chlamydia psittaci* and *Mycoplasma pneumoniae*) were analyzed in this study. *C. psittaci* and *M. pneumoniae* commonly cause pneumonia, while *B. alpica* is an aquatic bacterium first isolated in 1987 during outbreaks of pneumonia and meningitis that occurred in a spa therapy center [32]. Our IgM serological analysis shows that *B. alpica* was more frequently detected in the convalescent-phase sera than in admission sera (p* = *0.01). Antibodies against *B. alpica* were identified in five patients at their admission, but they were more frequent in convalescent sera; the high seroconversion rate (24% of patients) suggests that pneumonia patients in the ICU were commonly exposed to this bacterium. *M. pneumoniae* is a pathogen mainly associated with pneumonia episodes [33], [34]. In our patients, *M. pneumoniae* was identified in one serum from an unventilated HAP patient, as well as in two sera from two patients in a convalescent phase after VAP and CAP episodes. None of them had antibodies against this bacterium at admission, suggesting that this bacterium is widely associated with pneumonia. At last, none of the patients at admission or during their ICU stay had antibodies against *C. psittaci* which is known to be associated with pneumonia.

Another interesting result from this study is the high seroconversion rates for microorganisms associated with a prolonged ICU stay (Table 4). Patients who had a prolonged ICU stay had antibodies against higher number of microorganisms after their ICU stay compared to admission. For example, serum samples collected 21 days after admission had antibodies against up to 17 AAMs, which is concordant with previous studies showing that a longer ICU stay is associated with the development of VAP episodes. Moreover, our hierarchical cluster analysis showed that numerous pneumonia patients were exposed to a number of these AAMs, especially if they had a prolonged ICU stay (Fig.1 and 2).

Finally, it must be remembered that ICU patients are exposed to other waterborne and environmental bacteria widely associated to hospital-acquired infections, such as *Pseudomonas aeruginosa* and *Acinetobacter baumannii*, or to other pathogens from unknown origins. Consequently, the exposure of these patients is not limited to AAMs.

In summary, our work describes the magnitude of ICU patients exposure to amoeba-associated microorganisms, which have been increasingly isolated from hospital water sources and have been repeatedly reported to be associated with pneumonia. More in pneumonia, our work partially confirms previous studies showing ICU patients exposure to a number of these water AAMs, including *Afipia* genospecies 3, *Balneatrix alpica, Bosea eneae, Chlamydia pneumoniae* and Mimivirus. Moreover, our work highlights the importance of ICU patient exposure to AAMs, especially for patients that have a prolonged ICU stay. In contrast, we showed that other kinds of AAMs are ubiquitously detected and their role in hospital-acquired infections is not clear.

## Materials and Methods

### Patients and Clinical Specimens

All patients were from a cohort prospective study in three intensive care units (ICUs) in Marseille, France (one medical ICU and two medico-surgical ICUs) over a one-year period (from February 2007 until January 2008). The patient’s ages ranged from 18 to 94 years. Informed consent was obtained from the patients’ family members. The project was approved by the Local Ethics Committee (Marseille, France) and the permit number was 07–026. Blood samples were obtained from these patients at the admission in the ICU (control serum samples), when patients presented pneumonia symptoms (pneumonia serum samples or acute phase serum samples) and/or every 7 days following their admission to the ICU (weekly serum samples). Weekly serum samples performed after a pneumonia episode was considered to be a convalescent-phase serum samples. Ventilator-associated pneumonia, community-acquired pneumonia and aspiration pneumonia were diagnosed as previously described [30], [35], [36].

### Multiplexed Serologic Assay

We tested for the presence of serum antibodies against AAMs that have been previously isolated from hospital water sources and especially those AAMs which have been isolated from ICU patients with ventilator-associated pneumonia [8], [9], [13], [14], [37], [38]. More specifically, we tested patients serum samples for both IgG and IgM antibodies against *Afipia birgiae*, *Afipia broomeae, Bosea eneae, Afipia felis, Afipia felis* genospecies A, *Afipia* genospecies 1, *Afipia* genospecies 2, *Afipia* genospecies 3, *Afipia massiliae, Afipia quartiernordensis, Afipia saintantoinensis, Azorhizobium caulinodans, Bosea massiliensis, Bosea thiooxidans, Bosea vestrisii, Bradyrhizobium japonicum, Bradyrhizobium liaoningense, Mesorhizobium amorphae,* Mimivirus, *Nordella oligomobilis, Parachlamydia Acanthamoeba* (BN9) and Rasbo bacterium. Multiplexed serology was performed using amoeba-associated microorganism slides (Inodiag, Signe, France). Slides also targeted 5 non-AAMs (*Afipia clevelandensis, Balneatrix alpica, Chlamydia pneumoniae, Chlamydia psittaci* and *Mycoplasma pneumoniae*). Among them, *A. clevelandensis* and *B. alpica* were tested for both IgG and IgM antibodies, whereas *C. pneumoniae, C. psittaci* and *M. pneumoniae* were only tested for IgG or IgM antibodies respectively (Table S2).

Antibody titers were automatically determined and expressed as arbitrary fluorescence unit values (AUV). All cut-off values used in the present study were pre-programmed into the “InoSoft 1.4.4.4” software that was supplied with the slides. These cut-off values were determined as previously reported [17]. For antigens that had one cut-off value, the serum was considered positive if the fluorescence value was higher than the cut-off value. For antigens that had two cut-off values, the serum was considered positive if the fluorescence value was higher than the second cut-off value or low positive if the fluorescence value was between the first and second cut-off value. Cut-off values are listed in Table S2. The seroconversion rate for antibodies against these AAMs was assessed for patients for whom at least two serial serum samples were available (n* = *71). Seroconversion for each microorganism was defined as an increase in the arbitrary unit value (AUV) of the antibody titer from below the cut-off (or the first cut-off) in the first sample to above this cut-off (or higher than the second cut-off) in the second sample (Table S2). A serological reaction was performed on the patient serum samples as previously described [17], [19], [20]. The assay is fully automated.

### Hierarchical Clustering Analysis and Statistical Analysis

The final results for all of the serum samples (both IgG and IgM antibodies) were hierarchically clustered with the T-Mev software (www.tm4.org/mev.html) [39].

Statistical analysis was performed with a Chi-square test. P-values less than or equal to 0.05 were considered to be significant. All statistical tests have been done without correction for multiple statistical comparisons due to the fact that the importance of such statistical correction for multiple tests remains paradoxical [40]–[42].

## Supporting Information

Table S1 IgM to IgG class switching observation in patients with and without pneumonia.
(DOCX)

Table S2 The cut-off values that were used in this study. The cut-off values are expressed as arbitrary fluorescence unit values (AUV).
(DOC)

## References

[1] Gross PA, Neu HC, Aswapokee P, Van AC, Aswapokee N (1980) Deaths from nosocomial infections: experience in a university hospital and a community hospital. Am J Med 68: 219–223. 7355892 10.1016/0002-9343(80)90357-5

[2] Bueno-Cavanillas A, gado-Rodriguez M, Lopez-Luque A, Schaffino-Cano S, Galvez-Vargas R (1994) Influence of nosocomial infection on mortality rate in an intensive care unit. Crit Care Med 22: 55–60. 8124975 10.1097/00003246-199401000-00013

[3] Vincent JL, Bihari DJ, Suter PM, Bruining HA, White J, et al (1995) The prevalence of nosocomial infection in intensive care units in Europe. Results of the European Prevalence of Infection in Intensive Care (EPIC) Study. EPIC International Advisory Committee. JAMA 274: 639–644. 7637145

[4] Guidelines for the management of adults with hospital-acquired, ventilator-associated, and healthcare-associated pneumonia (2005) Am J Respir Crit Care Med. 171: 388–416. 10.1164/rccm.200405-644ST 15699079

[5] Greub G (2009) Parachlamydia acanthamoebae, an emerging agent of pneumonia. Clin Microbiol Infect 15: 18–28. 19220336 10.1111/j.1469-0691.2008.02633.x

[6] Raoult D, Renesto P, Brouqui P (2006) Laboratory infection of a technician by mimivirus. Ann Intern Med 144: 702–703. 10.7326/0003-4819-144-9-200605020-00025 16670147

[7] Berger P, Papazian L, Drancourt M, La Scola B, Auffray JP, et al (2006) Ameba-associated microorganisms and diagnosis of nosocomial pneumonia. Emerg Infect Dis 12: 248–255. 16494750 10.3201/eid1202.050434 PMC3373093

[8] La Scola B, Marrie TJ, Auffray JP, Raoult D (2005) Mimivirus in pneumonia patients. Emerg Infect Dis 11: 449–452. 15757563 10.3201/eid1103.040538 PMC3298252

[9] La Scola B, Boyadjiev I, Greub G, Khamis A, Martin C, et al (2003) Amoeba-resisting bacteria and ventilator-associated pneumonia. Emerg Infect Dis 9: 815–821. 12890321 10.3201/eid0907.030065 PMC3023432

[10] Greub G, Raoult D (2002) Parachlamydiaceae: potential emerging pathogens. Emerg Infect Dis 8: 625–630. 12023921 10.3201/eid0806.010210 PMC2738484

[11] Pagnier I, Raoult D, La Scola B (2008) Isolation and identification of amoeba-resisting bacteria from water in human environment by using an Acanthamoeba polyphaga co-culture procedure. Environ Microbiol 10: 1135–1144. 18279351 10.1111/j.1462-2920.2007.01530.x

[12] Thomas V, Herrera-Rimann K, Blanc DS, Greub G (2006) Biodiversity of amoebae and amoeba-resisting bacteria in a hospital water network. Appl Environ Microbiol 72: 2428–2438. 16597941 10.1128/AEM.72.4.2428-2438.2006 PMC1449017

[13] La Scola B, Mezi L, Auffray JP, Berland Y, Raoult D (2002) Patients in the intensive care unit are exposed to amoeba-associated pathogens. Infect Control Hosp Epidemiol 23: 462–465. 12186213 10.1086/502086

[14] La Scola B, Mallet MN, Grimont PA, Raoult D (2003) Bosea eneae sp. nov., Bosea massiliensis sp. nov. and Bosea vestrisii sp. nov., isolated from hospital water supplies, and emendation of the genus Bosea (Das et al. 1996). Int J Syst Evol Microbiol 53: 15–20. 12656146 10.1099/ijs.0.02127-0

[15] Mezzasoma L, Bacarese-Hamilton T, Di CM, Rossi R, Bistoni F, et al (2002) Antigen microarrays for serodiagnosis of infectious diseases. Clin Chem 48: 121–130. 11751547

[16] Gouriet F, Drancourt M, Raoult D (2006) Multiplexed serology in atypical bacterial pneumonia. Ann N Y Acad Sci 1078: 530–540. 17114771 10.1196/annals.1374.104

[17] Gouriet F, Levy PY, Samson L, Drancourt M, Raoult D (2008) Comparison of the new InoDiag automated fluorescence multiplexed antigen microarray to the reference technique in the serodiagnosis of atypical bacterial pneumonia. Clin Microbiol Infect 14: 1119–1127. 19076843 10.1111/j.1469-0691.2008.02119.x

[18] Gouriet F, Samson L, Delaage M, Mainardi JL, Meconi S, et al (2008) Multiplexed whole bacterial antigen microarray, a new format for the automation of serodiagnosis: the culture-negative endocarditis paradigm. Clin Microbiol Infect 14: 1112–1118. 19076842 10.1111/j.1469-0691.2008.02094.x

[19] Bonhomme CJ, Renesto P, Nandi S, Lynn AM, Raoult D (2008) Serological microarray for a paradoxical diagnostic of Whipple’s disease. Eur J Clin Microbiol Infect Dis 27: 959–968. 18594884 10.1007/s10096-008-0528-0

[20] Bonhomme CJ, Nappez C, Raoult D (2007) Microarray for serotyping of Bartonella species. BMC Microbiol 7: 59. 17593301 10.1186/1471-2180-7-59 PMC3225882

[21] Martins TB, Jaskowski TD, Tebo A, Hill HR (2009) Development of a multiplexed fluorescent immunoassay for the quantitation of antibody responses to four Neisseria meningitidis serogroups. J Immunol Methods 342: 98–105. 19159627 10.1016/j.jim.2008.12.003

[22] Martins TB, Welch RJ, Hill HR, Litwin CM (2009) Comparison of a multiplexed herpes simplex virus type-specific immunoglobulin G serology assay to immunoblot, Western blot, and enzyme-linked immunosorbent assays. Clin Vaccine Immunol 16: 55–60. 19020107 10.1128/CVI.00351-08 PMC2620677

[23] Casson N, Entenza JM, Greub G (2007) Serological cross-reactivity between different Chlamydia-like organisms. J Clin Microbiol 45: 234–236. 17065263 10.1128/JCM.01867-06 PMC1828948

[24] Birtles RJ, Rowbotham TJ, Storey C, Marrie TJ, Raoult D (1997) Chlamydia-like obligate parasite of free-living amoebae. Lancet 349: 925–926. 9093261 10.1016/s0140-6736(05)62701-8

[25] Greub G, Boyadjiev I, La Scola B, Raoult D, Martin C (2003) Serological hint suggesting that Parachlamydiaceae are agents of pneumonia in polytraumatized intensive care patients. Ann N Y Acad Sci 990: 311–319. 12860644 10.1111/j.1749-6632.2003.tb07381.x

[26] Lieberman D, Kahane S, Lieberman D, Friedman MG (1997) Pneumonia with serological evidence of acute infection with the Chlamydia-like microorganism “Z”. Am J Respir Crit Care Med 156: 578–582. 9279243 10.1164/ajrccm.156.2.9608081

[27] Marrie TJ, Raoult D, La Scola B, Birtles RJ, de CE (2001) Legionella-like and other amoebal pathogens as agents of community-acquired pneumonia. Emerg Infect Dis 7: 1026–1029. 11747734 10.3201/eid0706.010619 PMC2631911

[28] La Scola B, Campocasso A, N’Dong R, Fournous G, Barrassi L, et al (2010) Tentative characterization of new environmental giant viruses by MALDI-TOF mass spectrometry. Intervirology 53: 344–353. 20551686 10.1159/000312919

[29] Vincent A, La Scola B, Papazian L (2010) Advances in Mimivirus pathogenicity. Intervirology 53: 304–309. 20551682 10.1159/000312915

[30] Vincent A, La Scola B, Forel JM, Pauly V, Raoult D, et al (2009) Clinical significance of a positive serology for mimivirus in patients presenting a suspicion of ventilator-associated pneumonia. Crit Care Med 37: 111–118. 19050618 10.1097/CCM.0b013e318192fa8b

[31] Khan M, La Scola B, Lepidi H, Raoult D (2007) Pneumonia in mice inoculated experimentally with Acanthamoeba polyphaga mimivirus. Microb Pathog 42: 56–61. 17188457 10.1016/j.micpath.2006.08.004

[32] Dauga C, Gillis M, Vandamme P, Ageron E, Grimont F, et al (1993) Balneatrix alpica gen. nov., sp. nov., a bacterium associated with pneumonia and meningitis in a spa therapy center. Res Microbiol 144: 35–46. 8327781 10.1016/0923-2508(93)90213-l

[33] Takahashi T, Morozumi M, Okada T, Chiba N, Asami R, et al (2009) Prolonged Mycoplasma pneumoniae infection in an elderly patient with community-acquired pneumonia. J Infect Chemother 15: 243–247. 19688244 10.1007/s10156-009-0692-x

[34] Higashigawa M, Kawasaki Y, Yodoya N, Omori Y, Nashida Yet al (2009) Prevalence of Mycoplasma IgM in children with lower respiratory tract illness. Pediatr Int 51: 684–686. 19419518 10.1111/j.1442-200X.2009.02858.x

[35] Marik PE (2001) Aspiration pneumonitis and aspiration pneumonia. N Engl J Med 344: 665–671. 11228282 10.1056/NEJM200103013440908

[36] Mandell LA, Wunderink RG, Anzueto A, Bartlett JG, Campbell GD, et al (2007) Infectious Diseases Society of America/American Thoracic Society consensus guidelines on the management of community-acquired pneumonia in adults. Bull Johns Hopkins Hosp 44 Suppl 2S27–S72. 10.1086/511159 PMC7107997 17278083

[37] La Scola B, Fournier PE, Gouin F, Motte A, Raoult D (2005) Mycoplasma pneumoniae: a rarely diagnosed agent in ventilator-acquired pneumonia. J Hosp Infect 59: 74–75. 15571860 10.1016/j.jhin.2004.07.011

[38] La Scola B, Mallet MN, Grimont PA, Raoult D (2002) Description of Afipia birgiae sp. nov. and Afipia massiliensis sp. nov. and recognition of Afipia felis genospecies A. Int J Syst Evol Microbiol 52: 1773–1782. 12361286 10.1099/00207713-52-5-1773

[39] Saeed AI, Sharov V, White J, Li J, Liang W et al. TM4: a free, open-source system for microarray data management and analysis (2003) Biotechniques. 34: 374–8. 10.2144/03342mt01 12613259

[40] Thomas V Perneger. What’s wrong with Bonferroni adjustments (1998) British Medical Journal; Apr 18; 316, 7139. 10.1136/bmj.316.7139.1236 PMC1112991 9553006

[41] Nakagawa S A farewell to Bonferroni: the problems of low statistical power and publication bias (2004) Behav Ecol. 15: 1044–1045.

[42] Moran M.D (2003) Arguments for rejecting the sequential Bonferroni in ecological studies Oikos Volume 100, Issue 2, pages 403–405.

