# Supplementary material for: Serologic Prevalence of Amoeba-Associated Microorganisms in Intensive Care Unit Pneumonia Patients
Source: PLoS One. 2013 Mar 1;8(3):e58111. doi: 10.1371/journal.pone.0058111 (PMC3585915; doi:10.1371/journal.pone.0058111)
Supplement: Table S1 — IgM to IgG class switching observation in patients with and without pneumonia. (DOCX) [file pone.0058111.s001.docx]

Table S1: IgM to IgG class switching observation in patients with and without pneumonia

| Antigen | IgM to IgG class switching observation | | | | | | | |
| --- | --- | --- | --- | --- | --- | --- | --- | --- |
|  | CAP patients (n=9) | | HAP patients (n=48) | | All pneumonia patients (n=57) | | Patients without pneumonia (n=14) | |
| AAMs |  |  |  |  |  |  |  |  |
| Water Alpha-Proteobacteria : |  |  |  |  |  |  |  |  |
| *Afipia birgiae* | 0 |  | 0 |  | 0 |  | 0 |  |
| *Afipia broomeae* | 0 |  | 0 |  | 0 |  | 0 |  |
| *Afipia felis* | 0 |  | 6 |  | 6 |  | 1 |  |
| *Afipia felis* genospecies A | 2 |  | 3 |  | 5 |  | 0 |  |
| *Afipia* genospecies 1 | 0 |  | 0 |  | 0 |  | 0 |  |
| *Afipia* genospecies 2 | 0 |  | 1 |  | 1 |  | 0 |  |
| *Afipia* genospecies 3 | 1 |  | 3 |  | 4 |  | 1 |  |
| *Afipia massiliae* | 0 |  | 0 |  | 0 |  | 0 |  |
| *Afipia quartiernordensis* | 0 |  | 0 |  | 0 |  | 1 |  |
| *Afipia saintantoinensis* | 0 |  | 0 |  | 0 |  | 0 |  |
| *Azorhizobium caulinodans* | 0 |  | 1 |  | 1 |  | 0 |  |
| *Bosea eneae* | 0 |  | 1 |  | 1 |  | 0 |  |
| *Bosea massiliensis* | 0 |  | 2 |  | 2 |  | 0 |  |
| *Bosea thiooxidans* | 0 |  | 0 |  | 0 |  | 1 |  |
| *Bosea vestrisii* | 1 |  | 0 |  | 1 |  | 0 |  |
| *Bradyrhizobium japonicum* | 2 |  | 1 |  | 3 |  | 1 |  |
| *Bradyrhizobium liaoningense* | 1 |  | 9 |  | 10 |  | 2 |  |
| *Mesorhizobium amorphae* |  |  |  |  | 0 |  | 0 |  |
| *Nordella oligomobilis* | 2 |  | 0 |  | 2 |  | 0 |  |
| Rasbo bacterium | 0 |  | 0 |  | 0 |  | 0 |  |
| Chlamydiae: |  |  |  |  |  |  |  |  |
| *Parachlamydia acanthamoeba* BN9 | 0 |  | 6 |  | 6 |  | 2 |  |
| Water viruses: |  |  |  |  |  |  |  |  |
| Mimivirus | 2 |  | 5 |  | 7 |  | 0 |  |
| Non-AAMs |  |  |  |  |  |  |  |  |
| *Afipia clevelandensis* | 0 |  | 0 |  | 0 |  | 0 |  |
| *Balneatrix alpica* | 2 |  | 3 |  | 5 |  | 0 |  |
| *Chlamydia pneumoniae* | ND |  | ND |  | ND |  | ND |  |
| *Chlamydia psittaci* | ND |  | ND |  | ND |  | ND |  |
| *Mycoplasma pneumoniae* | ND |  | ND |  | ND |  | ND |  |

CAP, community-acquired pneumonia; HAP, hospital-acquired pneumonia ; ND, not determined.
