# Supplementary material for: Serologic Prevalence of Amoeba-Associated Microorganisms in Intensive Care Unit Pneumonia Patients
Source: PLoS One. 2013 Mar 1;8(3):e58111. doi: 10.1371/journal.pone.0058111 (PMC3585915; doi:10.1371/journal.pone.0058111)
Supplement: Table S2 — The cut-off values that were used in this study. The cut-off values are expressed as arbitrary fluorescence unit values (AUV). (DOC) [file pone.0058111.s002.doc]

Table S2: The cut-off values that were used in this study. The cut-off values are expressed as arbitrary fluorescence unit values (AUV).

| Antigen | IgG | | IgM | |
| --- | --- | --- | --- | --- |
|  | Cut-off 1 (AUV) | Cut-off 2 (AUV) | Cut-off 1 (AUV) | Cut-off 2 (AUV) |
| *Afipia birgiae* | 3000 | No | 3000 | No |
| *Afipia broomeae* | 3500 | No | 3500 | No |
| *Afipia clevelandensis* | 4000 | No | 4500 | No |
| *Afipia felis* | 3500 | No | 3500 | No |
| *Afipia felis* genospecies A | 5000 | No | 4000 | No |
| *Afipia* genospecies 1 | 2000 | No | 2000 | No |
| *Afipia* genospecies 2 | 3000 | No | 3000 | No |
| *Afipia* genospecies 3 | 6000 | No | 3000 | No |
| *Afipia massiliae* | 5000 | No | 3500 | No |
| *Afipia quartiernordensis* | 2500 | No | 3500 | No |
| *Afipia saintantoinensis* | 4000 | No | 5000 | No |
| *Azorhizobium caulinodans* | 5500 | No | 2500 | No |
| *Balneatrix alpica* | 2000 | No | 2000 | No |
| *Bosea eneae* | 2000 | No | 2000 | No |
| *Bosea massiliensis* | 1500 | No | 1500 | No |
| *Bosea thioxydans* | 1500 | No | 1000 | No |
| *Bosea vestrisii* | 800 | No | 1300 | No |
| *Bradyrhizobium japonicum* | 1500 | No | 5000 | No |
| *Bradyrhizobium liaoningense* | 1000 | No | 3500 | No |
| *Chlamydia pneumoniae* | 500 | 800 | NT | No |
| *Chlamydia psittaci* | 1000 | 1500 | NT | No |
| *Mesorhizobium amorphae* | 2000 | No | 2500 | No |
| *Mycoplasma pneumoniae* | NT | No | 1000 | 2000 |
| Mimivirus | 900 | 1800 | 900 | 1800 |
| *Nordella oligomobilis* | 3000 | No | 3000 | No |
| *Parachlamydia acanthamoeba* BN9 | 1000 | No | 1000 | No |
| *Rasbo bacterium* | 2000 | No | 1500 | No |

NT; not tested
